# Antimicrobial Resistance and Genomic Characterization of a Novel ST181 *Streptococcus parasuis* Clinical Isolate from Human Pleural Fluid

**DOI:** 10.3390/antibiotics15090893

**Published:** 2026-09-11

**Authors:** Zhenghao Jie, Hongyu Lei, Yuhui Tian, Fekadu Gutema Wegi, Weijiang Liu, Long Ye, Xiaojun Tan, Jingfang Zhou, Jiayi Pan, Shaofang Lin, Pishun Li, Xiaofeng Zheng, Xuxia Cui

**Affiliations:** 1 College of Veterinary Medicine and Hunan Engineering Technology Research Center of Veterinary Drugs, Hunan Agricultural University, Changsha 410128, China; 2 Holeta Agricultural Research Center, Ethiopian Institute of Agricultural Research, Addis Ababa 2003, Ethiopia; 3 Laboratory Medicine, Guangdong Provincial People’s Hospital (Guangdong Academy of Medical Sciences), Southern Medical University, Guangzhou 510080, China

**Keywords:** *Streptococcus parasuis*, antimicrobial resistance, ST181, comparative genomics, *Galleria mellonella*

## Abstract

**Background/Objectives:** *Streptococcus parasuis* is an under-recognized member of the *Streptococcus suis* complex that can be misidentified by routine diagnostic methods. Human clinical isolates remain sparsely characterized. This study investigated the antimicrobial phenotype, genomic features, population position, and larval pathogenicity of a human pleural-fluid isolate. **Methods:** Strain HUGSPH was recovered during the care of a patient with pneumonia. Hybrid whole-genome sequencing (WGS), average nucleotide identity analysis, multilocus sequence typing (MLST), core-genome phylogenetics, comparative pan-genomics, and an exploratory host-origin association analysis were performed. Minimum inhibitory concentrations (MICs) were determined using the VITEK 2 Compact system with an AST-ST03 card, and virulence was evaluated in *Galleria mellonella* larvae. **Results:** Genome-based analysis confirmed HUGSPH as *S. parasuis* and assigned it to the novel sequence type (ST) 181. The isolate clustered within a predominantly human-origin phylogenetic clade. Because species-specific clinical breakpoints are unavailable, the MICs were interpreted provisionally using explicitly stated surrogate Clinical and Laboratory Standards Institute (CLSI) criteria; erythromycin, clindamycin, and levofloxacin were categorized as resistant under those criteria. Several predicted antimicrobial-resistance-associated genes were detected. In a restricted comparison of seven human-origin and nine swine-origin genomes, eight genes were present in all included human-origin isolates and absent from all included swine-origin isolates, whereas *metQ*, *metP*, and *metN* showed the reciprocal pattern. HUGSPH caused dose-dependent larval mortality, reaching 100% at 10^7^ colony-forming units (CFU) by 96 h. **Conclusions:** HUGSPH expands the genomic record of human clinical *S. parasuis* and highlights the diagnostic and surveillance relevance of antimicrobial resistance in this species. The susceptibility categories, source-associated genes, and larval phenotype require validation by reference susceptibility testing, broader phylogenetically balanced collections, comparative strains, and mammalian models.

## 1. Introduction

*Streptococcus suis* is a swine zoonotic pathogen that causes severe invasive disease, meningitis and septicemia in both swine and humans worldwide [1]. Transmission typically occurs through contact with infected swine or consumption of contaminated pork products [2,3]. Recent genomic revisions have reshaped the taxonomy of this group. In 2015, combined biochemical characterization, 16S rRNA sequencing, and average nucleotide identity (ANI) analysis led to the reclassification of serotypes 20, 22, and 26 as a new species, *Streptococcus parasuis* [4]. Although *S. parasuis* has been recovered from both healthy and diseased swine and cattle, its pathogenic potential has long been overshadowed by diagnostic limitations [5,6].

Routine phenotypic testing and matrix-assisted laser desorption/ionization time-of-flight mass spectrometry (MALDI-TOF MS) frequently misidentify *S. parasuis* as *S. suis* because reference spectra are incomplete and the two species overlap morphologically [7,8]. A recent genomic re-evaluation of isolates previously labeled as *S. suis* found that a substantial proportion were actually *S. parasuis* or other closely related species [9]. Over the past several years, at least eight provinces in China have reported at least 11 laboratory-confirmed human cases of *S. parasuis* infection, presenting as pneumonia, peritonitis, arthritis, and sepsis [10,11,12]. A case in Henan Province is especially notable: a sanitation worker with no documented livestock contact developed the infection, suggesting that environmental exposure, through contaminated soil, water, or fomites, may also be relevant [13,14].

Genomic epidemiology has begun to clarify the population structure of *S. parasuis*. An analysis of 255 genomes assigned the species into 12 minimum core genome (MCG) clusters and 161 sequence types (STs) under a newly developed multilocus sequence typing (MLST) scheme [15]. Known human-derived clinical isolates fall within MCG cluster 2, indicating source-associated population structure in the available dataset, although clustering alone does not establish host adaptation [16]. The species has an open pan-genome, indicating high genetic plasticity and extensive horizontal gene transfer that facilitate the acquisition of antimicrobial resistance (AMR) genes [6]. Mobile genetic elements such as integrative and conjugative elements (ICEs) disseminate clinically important resistance genes, including *optrA* and *cfr(D)* [17,18]. Moreover, *S. parasuis* shows a pathobiological profile distinct from that of *S. suis*: rather than the pronounced neurotropism and blood–brain barrier disruption seen with *S. suis*, *S. parasuis* predominantly targets peripheral organs such as the liver and lungs in mouse models [11,19].

Despite growing recognition of *S. parasuis* in human clinical infections, the genomic diversity of human-origin isolates remains incompletely described. Identifying novel STs, resistance-associated features, and candidate virulence-associated genes can inform epidemiological surveillance. Here, we report the isolation and genomic characterization of HUGSPH, an *S. parasuis* isolate recovered from the pleural fluid of a patient with pneumonia in Guangzhou, China. Through whole-genome sequencing (WGS), comparative genomics, a pan-genome-wide association study (pan-GWAS), and a larval infection model, we describe its genomic features, identify source-associated gene-content patterns, and assess dose-dependent larval mortality.

## 2. Results

### 2.1. Isolation and Preliminary Identification

**Figure 1:**
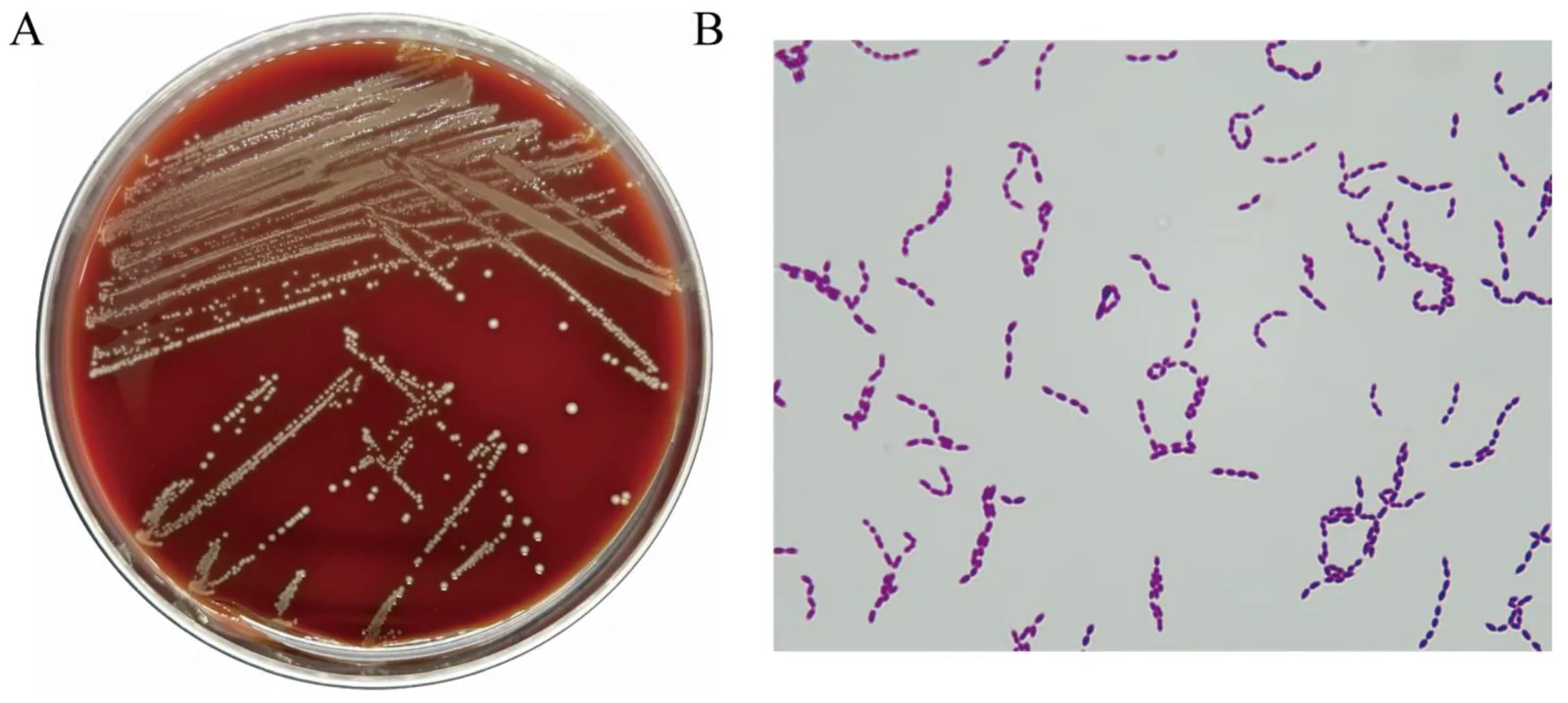
Morphological and microscopic characteristics of the purified clinical isolate HUGSPH. (**A**) Morphologically uniform colonies on 5% sheep blood agar after single-colony purification. (**B**) Gram-stained smear examined by light microscopy at ×1000 magnification.

HUGSPH was isolated from the pleural fluid of a patient with pneumonia. On 5% sheep blood agar, the isolate formed grayish-white, opaque, circular, smooth, slightly convex colonies surrounded by a greenish zone of alpha-hemolysis (Figure 1A). Suspected colonies were purified by repeated single-colony streaking, producing morphologically uniform growth; a single colony from the purified culture was used for genome sequencing. Gram staining revealed Gram-positive cocci predominantly arranged in short chains (Figure 1B), consistent with the genus *Streptococcus*. VITEK MS initially identified the isolate as *S. suis*. The measured minimum inhibitory concentrations (MICs) and quality-control results are presented in Table 1. Under the stated surrogate criteria, HUGSPH was provisionally categorized as resistant to erythromycin, clindamycin, and levofloxacin.

### 2.2. Genomic Features, Species Confirmation, and Sequence Typing

Hybrid assembly of short- and long-read data yielded a complete, single circular chromosome of 1,926,750 bp (N50 = 1,926,750 bp) with a guanine–cytosine (GC) content of 40% and 1905 coding sequences. CheckM estimated 100% genome completeness and 0.05% contamination.

**Figure 2:**
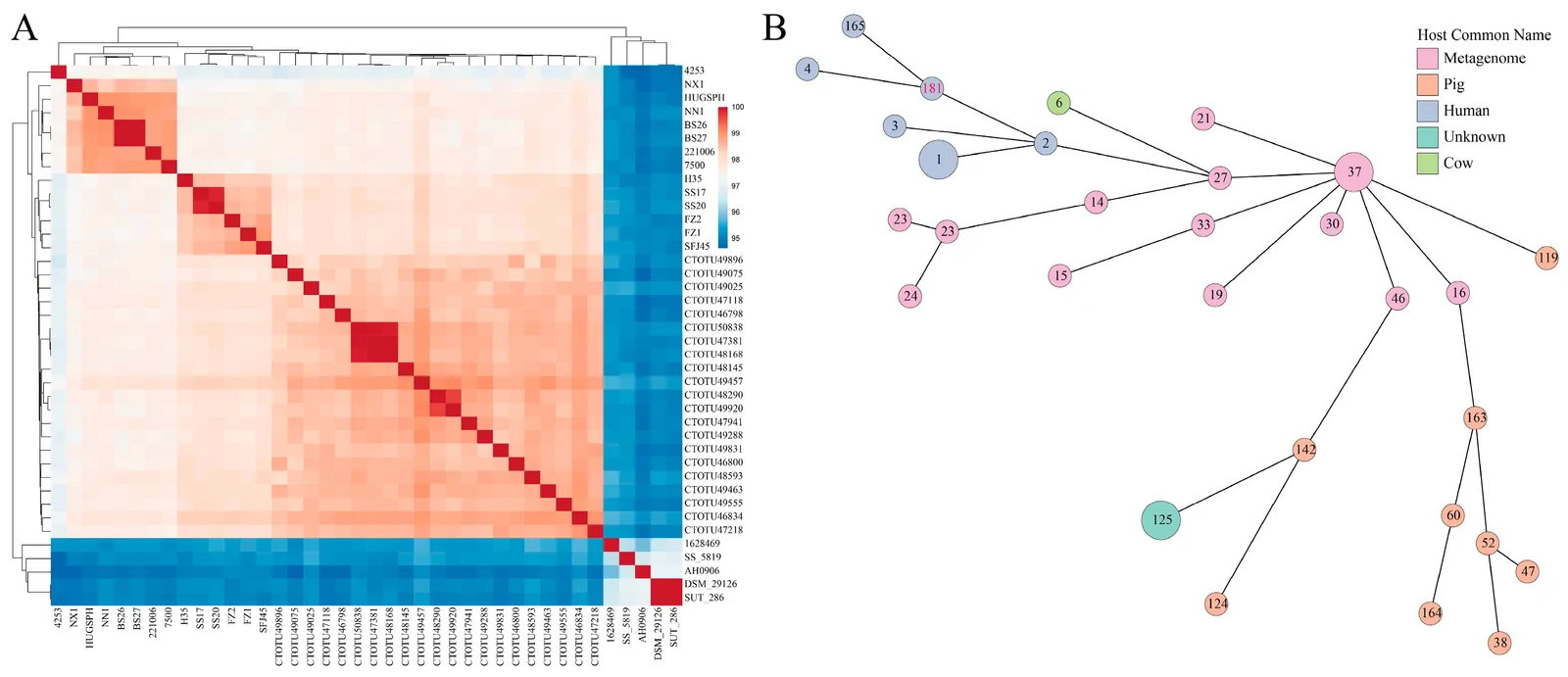
Genome-based species identification and sequence typing. (**A**) Hierarchical clustering heatmap of average nucleotide identity values between HUGSPH and 39 reference *S. parasuis* genomes; exact pairwise values are provided in Supplementary Table S1. (**B**) Minimum spanning tree based on multilocus sequence typing allelic profiles of publicly available *S. parasuis* isolates. Node colors denote host origin (blue, human; orange, pig; pink, metagenome; cyan, unknown; light green, cow). Node size is proportional to the number of isolates represented by each ST. The HUGSPH node is labeled ST181 and highlighted in red.

Mash v2.3 identified the isolate as *S. parasuis*. To confirm, we retrieved 39 reference *S. parasuis* genomes from the National Center for Biotechnology Information (NCBI). ANI analysis showed that HUGSPH shared >95% ANI with all reference genomes (range, 95.05–98.95%; Supplementary Table S1) and formed a distinct cluster within the *S. parasuis* clade (Figure 2A). MLST based on seven housekeeping genes (*aroA*, *cpn60*, *gki*, *mutS*, *recA*, *sdhA*, *thrA*) revealed a novel allelic profile, 1-41-1-3-2-4-37, which was officially assigned as ST181 upon submission to PubMLST. In the minimum spanning tree (Figure 2B), ST181 clustered within the human-origin clade, directly linked to ST165 and ST2. This human branch was separated from the large metagenome-origin cluster centered at ST37 and the main swine-origin clade in the lower right of the tree.

### 2.3. Core-Genome Phylogeny and Antimicrobial Resistance Gene Profile

**Figure 3:**
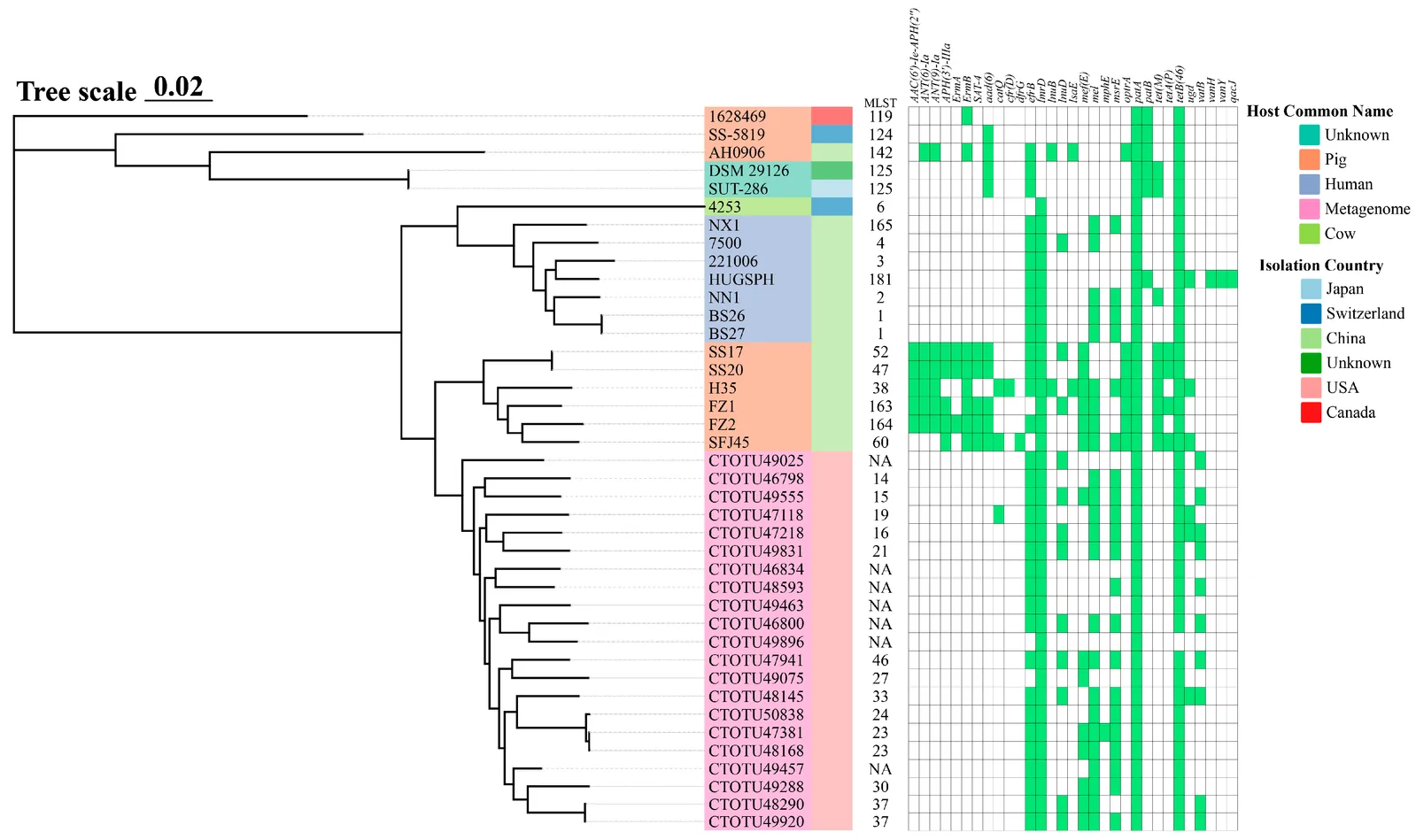
Core-genome phylogeny and predicted antimicrobial resistance gene profiles of *S. parasuis*. The maximum-likelihood tree was inferred from core-genome single-nucleotide polymorphisms after recombination filtering. Colored annotation strips denote host origin and country of isolation, the adjacent numeric column gives the multilocus sequence type, and the right-hand matrix indicates detected resistance-associated genes. HUGSPH (ST181) is shown within the predominantly human-origin clade.

A maximum-likelihood tree based on core-genome single-nucleotide polymorphisms (SNPs; recombinant regions removed with Gubbins) placed HUGSPH within a predominantly human-origin cluster (blue nodes in Figure 3) that included strains of ST1, ST2, ST3, ST4, and ST165 alongside HUGSPH itself (ST181). This clade was evolutionarily distinct from the swine-origin cluster at the top (ST119, ST124, ST142) and the metagenome-origin cluster at the bottom. HUGSPH carried the predicted resistance-associated genes *efrB*, *lmrD*, *patA*, *patB*, *tetB(46)*, *ugd*, *vanH*, *vanY*, and *qacJ*. Among these, *efrB*, *patA*, and *tetB(46)* were widely prevalent across the analyzed *S. parasuis* genomes. These sequence annotations predict gene presence but do not establish expression, functional resistance, or a complete resistance pathway.

### 2.4. Pan-Genome Analysis and Source-Associated Gene Content

**Figure 4:**
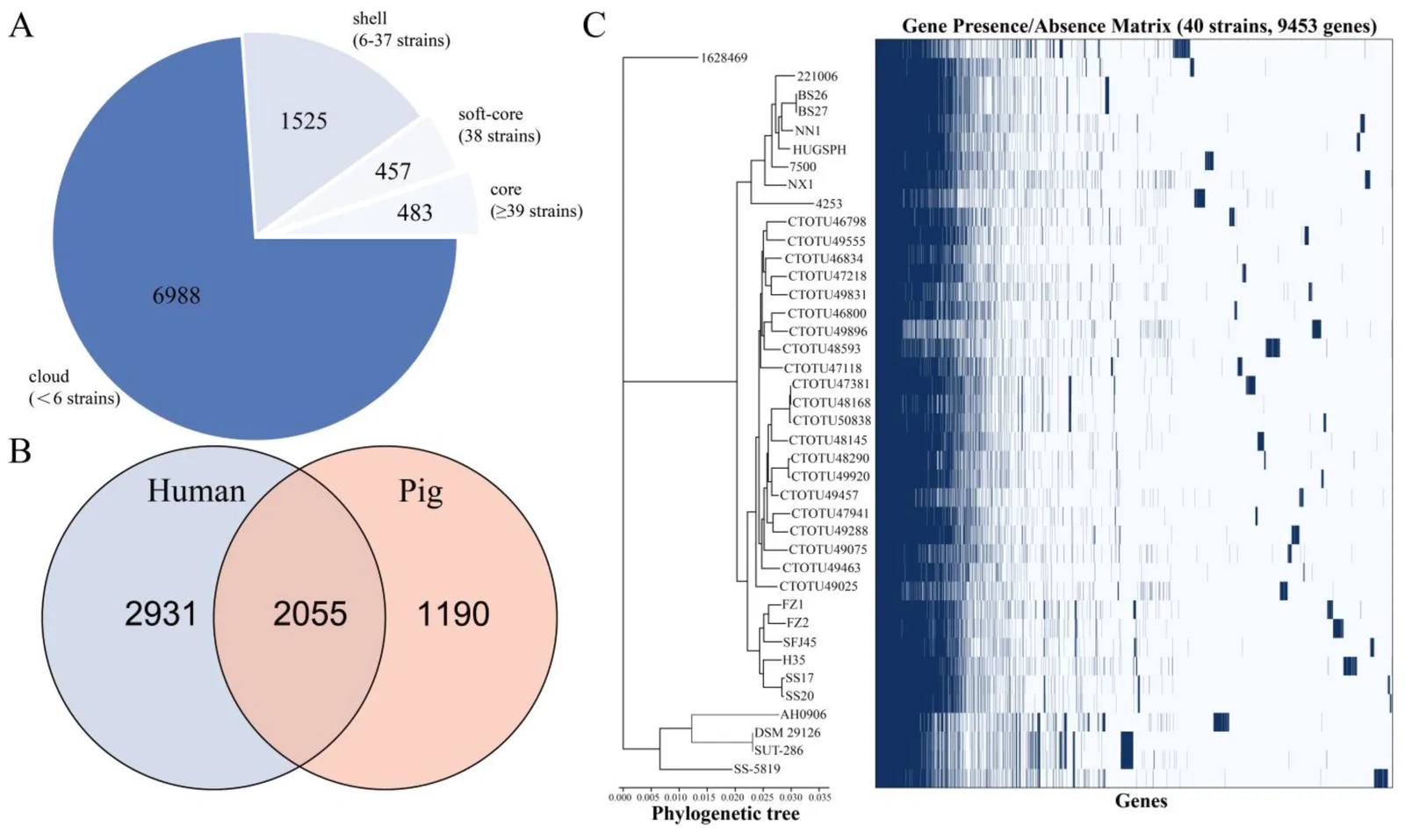
Pan-genome characteristics and source-associated genomic variation. (**A**) Composition of the *S. parasuis* pan-genome. (**B**) Numbers of shared and source-specific genes among seven human-origin and nine swine-origin isolates. (C) Gene presence–absence matrix for 40 S. parasuis genomes, comprising 9453 genes. In panel A, blue shades denote pan-genome categories; in panel B, blue and orange denote human- and swine-origin gene sets, respectively; in panel C, dark blue indicates gene presence and white indicates absence.

Pan-genome analysis of the 40 *S. parasuis* genomes identified 9453 genes in total. Of these, 483 were core genes (present in ≥39 strains), 457 were soft-core genes (present in 38 strains), 1525 were shell genes (present in 6–37 strains), and 6988 belonged to the accessory/cloud genome (present in <6 strains) (Figure 4A). Comparing the 7 human-origin and 9 swine-origin isolates, a Venn diagram showed 2055 shared genes, 2931 human-specific genes, and 1190 swine-specific genes (Figure 4B). The gene presence–absence matrix of all 40 strains is shown in Figure 4C.

A pan-genome-wide association study (pan-GWAS) with Scoary identified eight genes strictly associated with human isolates (100% presence in human, absent in swine; false discovery rate [FDR] *q* < 0.05): *yxdM*, *nrdE2*, *hcxA*, *yfkJ*, *yodC*, *nrdI*, *elaA*, and *nrdF* (Table 2). Three methionine import and metabolism genes, *metQ*, *metP*, and *metN*, were exclusively present in all swine isolates and completely absent in human isolates.

### 2.5. Comparative Genomic and Functional Analysis

**Figure 5:**
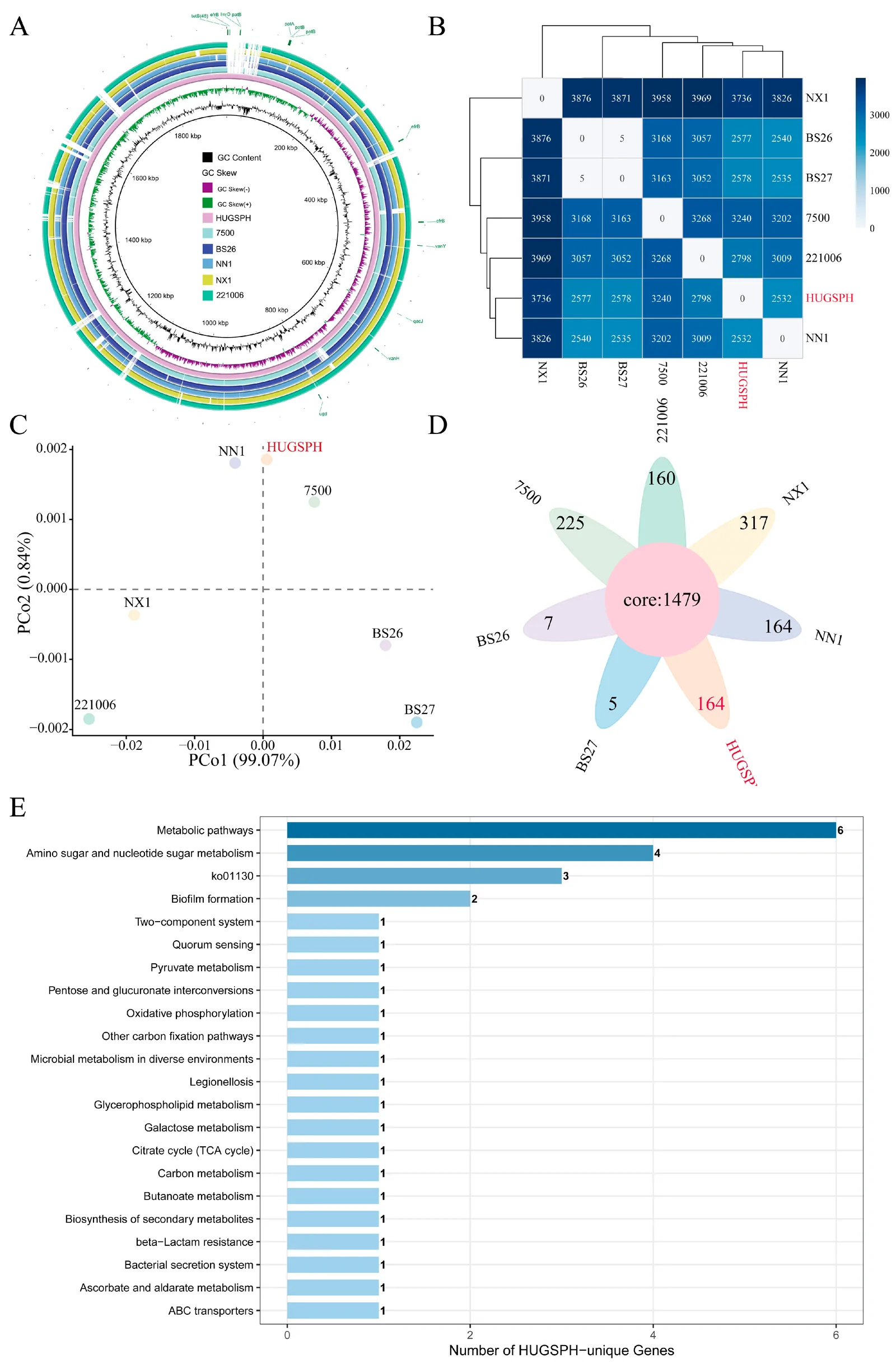
Comparative genomic and functional analysis of HUGSPH and six closely related *S. parasuis* genomes. (**A**) Circular genome alignment generated with the Basic Local Alignment Search Tool (BLAST) Ring Image Generator (BRIG) v0.95, with HUGSPH as the reference. (**B**) Pairwise gene-content distances. (**C**) Principal coordinate analysis of Clusters of Orthologous Genes presence-absence profiles. (**D**) Core and strain-specific gene repertoires. (**E**) KEGG category assignments among 164 genes unique to HUGSPH within the seven-genome comparison. In panels (**B**–**D**), labels or values for HUGSPH are shown in red; the dotted lines in panel (**C**) indicate zero values on the two coordinate axes.

Circular genome alignment showed overall structural conservation alongside distinct variable regions between HUGSPH and the reference genomes (Figure 5A). Gene-content-based pairwise distances placed HUGSPH closest to NN1 (distance 2532), with greater divergence from NX1 (3976) and 221,006 (3200) (Figure 5B). Principal coordinate analysis (PCoA) based on Clusters of Orthologous Genes (COG) profiles corroborated these relationships: the first two principal coordinates accounted for 99.91% of the total variance; HUGSPH clustered with NN1 and 7500, while other genomes dispersed along PC1 and PC2 (Figure 5C). The seven strains shared a core genome of 1479 genes. HUGSPH carried 164 unique genes, comparable to NN1 (164) and fewer than NX1 (317) and 7500 (225) (Figure 5D). Kyoto Encyclopedia of Genes and Genomes (KEGG) mapping assigned the HUGSPH-unique genes to several functional categories, with the largest numbers assigned to metabolic pathways (*n* = 6) and amino sugar and nucleotide sugar metabolism (*n* = 4), ko01130 (*n* = 3), biofilm formation (*n* = 2), and accessory pathways including two-component systems and quorum sensing (*n* = 1 each) (Figure 5E).

### 2.6. Virulence Gene Profile and Larval Pathogenicity

Screening against the Virulence Factor Database (VFDB) identified virulence-associated genes classified into adherence (*srtA*, *pavA*, *cbpD*, *plr/gapA*, *orf00559*, *orf01422*), enzymatic activity (*eno*), immune evasion (*cdsA*), protease activity (*htrA/degP*, *tig/ropA*), and cell surface components (*sugC, lgt*) (Table 3).

**Figure 6:**
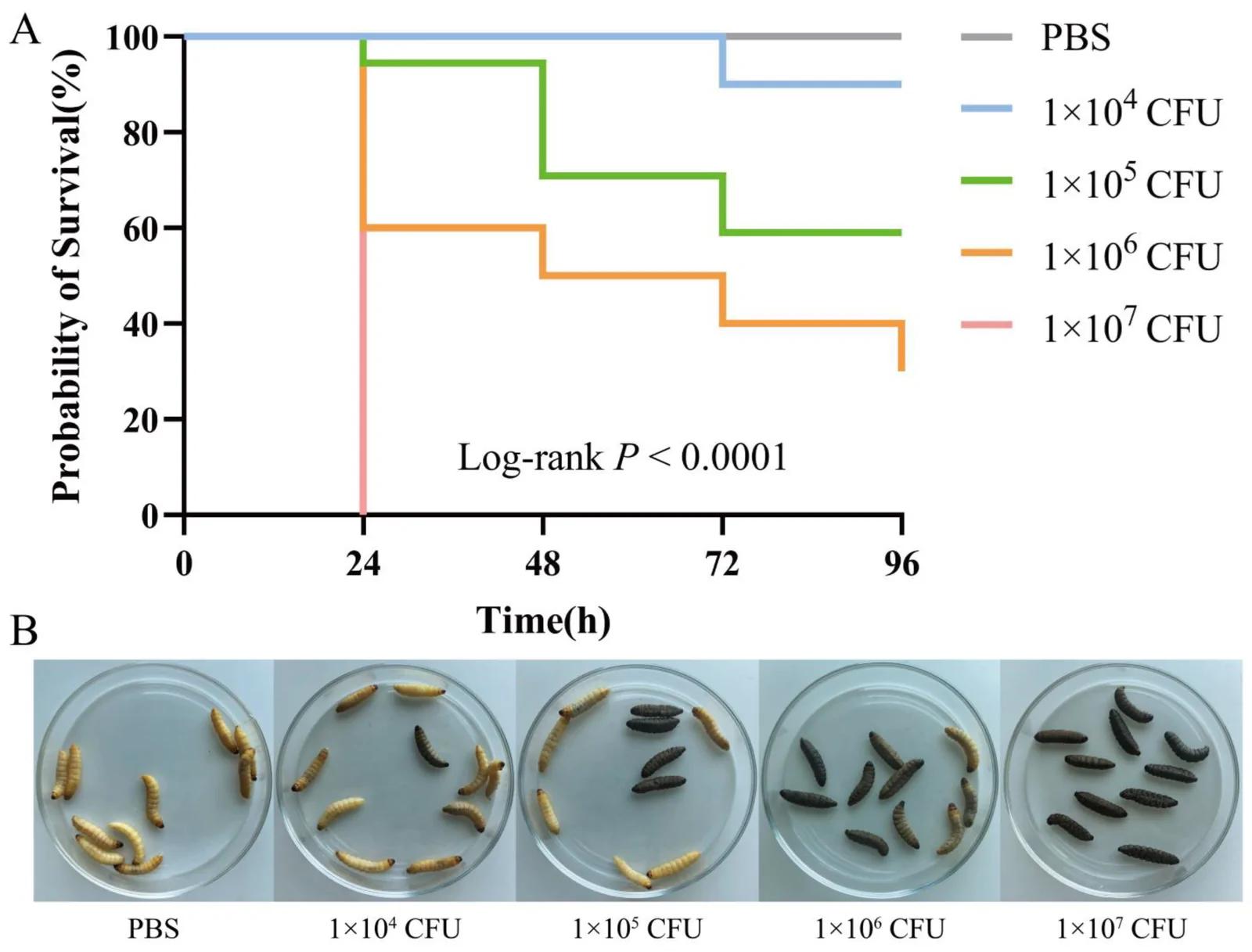
*In vivo* phenotype of HUGSPH in the *Galleria mellonella* larval model. (**A**) Kaplan–Meier survival curves for larvae (*n* = 10 per group) inoculated with 1 × 10^4^ to 1 × 10^7^ CFU of HUGSPH or PBS and monitored for 96 h. An untreated control group was monitored in parallel but is not plotted. (**B**) Representative photographs at 96 h post-inoculation. Larvae were considered dead when they showed melanization, lacked movement, and did not respond to touch.

In the *G. mellonella* larval model, doses from 10^4^ to 10^7^ colony-forming units (CFU) produced dose-dependent mortality over 96 h (log-rank test, χ^2^ = 50.70, *df* = 4, *p* < 0.0001) (Figure 6A). At 96 h, mortality was approximately 40% at 10^5^ CFU, 70% at 10^6^ CFU, and 100% at 10^7^ CFU (Figure 6B). All larvae in the phosphate-buffered saline (PBS)-injected and untreated control groups survived through 96 h.

## 3. Discussion

In this study, we characterized the *S. parasuis* isolate HUGSPH, which was recovered from pleural fluid during the care of a patient with pneumonia and assigned to the novel sequence type ST181. Its genome-resolved characterization, antimicrobial phenotype, and larval-mortality phenotype expand the available data for human clinical *S. parasuis* isolates. The phylogenetic and gene-content analyses provide source-associated observations within the included genome set, rather than direct evidence of host adaptation or transmission.

### 3.1. Phylogenetic and Population Structure

Accurate species-level identification of *S. parasuis* remains a diagnostic challenge for MALDI-TOF MS [7,8]. HUGSPH was initially identified as *S. suis*, whereas subsequent ANI analysis classified it as *S. parasuis*, with >95% similarity to the included reference genomes. This discordance supports improving reference-spectrum databases and applying genome-based confirmation where species-level assignment is clinically important [7]. Core-genome phylogeny placed HUGSPH in a branch containing predominantly human-origin genomes, including ST1, ST2, ST3, ST4, and ST165, and MLST assigned it to the novel ST181. This observation is consistent with the population-structure study by Zhang et al. (2025), in which available human clinical isolates were classified in MCG cluster 2 [15]. HUGSPH was assembled as a single circular chromosome, whereas a plasmid-bearing swine-origin strain A1 from Xinjiang has been described previously [21]. However, differences in genome content or structure alone do not establish adaptation to humans. Genome reduction can accompany host association in some bacterial lineages [22], but this possibility requires comparison across broader, phylogenetically balanced collections.

### 3.2. Antimicrobial Resistance at the One Health Interface

Using the explicitly stated surrogate criteria, HUGSPH was provisionally categorized as resistant to erythromycin, clindamycin, and levofloxacin. The erythromycin MIC (2 mg/L) was lower than the 8 mg/L reported for the *S. parasuis* isolates BS26 and BS27, whereas the HUGSPH trimethoprim–sulfamethoxazole MIC [≤0.5/9.5 mg/L (≤10 mg/L total)] was lower than the >32 mg/L total reported for those isolates [10]. These cross-study comparisons are descriptive because the test platforms and interpretive assumptions were not identical. Genome annotation identified several resistance-associated genes. *efrB*, *lmrD*, *patA*, and *patB* encode or are associated with efflux systems; the *patA*-*patB* system has been linked to reduced fluoroquinolone susceptibility in streptococci [23] and is therefore a candidate explanation for the levofloxacin phenotype. *tetB(46)* is a predicted tetracycline-resistance-associated determinant, although HUGSPH remained provisionally susceptible to tetracycline, illustrating genotype–phenotype discordance. *qacJ* is associated with efflux of quaternary-ammonium antiseptics rather than the antibiotics tested here. Importantly, detection of *vanH* and *vanY* homologues alone does not demonstrate a complete, functional vancomycin-resistance operon; the very low vancomycin MIC (≤0.12 mg/L) provided no phenotypic evidence of reduced susceptibility under the tested conditions. Gene detection cannot establish transcription, protein function, or causality. Published studies have documented diverse AMR gene profiles and mobile oxazolidinone-resistance determinants, including *optrA* and *cfr(D)*, in swine-origin *S. parasuis* [17,21,24,25,26,27]. HUGSPH did not carry these genes. *S. suis* likewise carries a diverse resistome [25], and a mobile *optrA*-carrying element from *S. parasuis* has been shown to transfer to *S. suis* [26]; however, direct gene-prevalence comparisons are not appropriate because the available studies differ in isolate composition and analytical methods. Continued genomic and phenotypic surveillance is warranted, but the provisional categories reported here require confirmation by a reference MIC method and future *S. parasuis*-specific interpretive criteria.

### 3.3. Source-Associated Gene-Content Patterns and Metabolic Hypotheses

Comparative pan-genomics and pan-GWAS identified eight genes present in all included human-origin isolates and absent from all included swine-origin isolates, whereas three genes were present in all included swine-origin isolates and absent from all included human-origin isolates. These findings identify candidate gene-content patterns for further investigation [28]. The *nrdE2* and *nrdI* genes encode components of ribonucleotide reductase, an enzyme system involved in deoxyribonucleic acid (DNA) synthesis and repair. Their distribution may be relevant to persistence in particular host environments, but this interpretation requires validation in broader, phylogenetically balanced datasets and by functional experiments. Conversely, the *metQNP* genes encode a methionine-uptake system that showed the reciprocal pattern in the included swine-origin isolates. Nutrient-acquisition systems can help bacterial pathogens compete with resident microbiota and establish colonization [29]. We did not evaluate the completeness of methionine-biosynthesis pathways in the human- and swine-origin genomes. Therefore, the observed absence of *metQNP* cannot be interpreted as methionine auxotrophy, and its association with the included swine-origin isolates remains a hypothesis-generating gene-content pattern rather than evidence that methionine metabolism determines host tropism.

### 3.4. Potential Virulence and Larval Pathogenicity

VFDB screening identified genes annotated as participating in adherence, enzymatic activity, immune evasion, protease activity, and cell-surface functions. In contrast to highly virulent *S. suis*, HUGSPH lacked the classical virulence markers *mrp*, *epf*, and *sly* [30]. This profile is compatible with previous descriptions of *S. parasuis* pathogenic strategies [31], but annotation alone does not establish gene expression or contribution to pathogenicity. In the *Galleria mellonella* model, HUGSPH caused dose-dependent mortality over 96 h, whereas both control groups survived. Although the lethal kinetics appeared slower than those reported for hypervirulent *S. suis* strains [11], increasing mortality with inoculum size is a nonspecific screening phenotype. Without comparator strains or targeted mutants, these data cannot rank HUGSPH virulence or attribute lethality to any predicted virulence gene. The experiment demonstrates larval killing under the tested conditions but does not establish human pathogenicity, tissue tropism, or a causal role for the annotated adhesins and proteases [32,33].

### 3.5. Limitations

First, this study is based on a single human clinical isolate; although WGS provided high-resolution genomic data, the findings cannot be generalized to the wider *S. parasuis* population. Second, no patient-linked animal or environmental samples were available, so potential environmental or indirect transmission routes could not be assessed. Third, the pan-GWAS comparison included limited and phylogenetically structured source-labeled groups, and the completeness of methionine-biosynthesis pathways was not examined. The functional roles of the associated genes, and whether their distributions are independent of lineage structure, remain unknown; broader balanced collections and gene knockout or complementation studies will be required. Fourth, MICs were generated using an automated VITEK 2 Compact AST-ST03 card, and *S. parasuis*-specific clinical breakpoints are unavailable. The surrogate categories are therefore exploratory and should not direct clinical treatment without confirmatory testing. Finally, the *Galleria mellonella* experiment used one cohort of 10 larvae per dose and lacked bacterial comparator strains and targeted mutants. It demonstrates dose-associated larval lethality but cannot quantify relative virulence, identify specific determinants, or reproduce mammalian immune responses and tissue tropism.

### 3.6. Implications for Surveillance and Public Health

Human *S. parasuis* infections, including cases without documented livestock contact, justify evaluation of environmental or indirect exposure routes in future studies [14,34]. Although HUGSPH was recovered during the care of a patient with pneumonia in Guangzhou and grouped with predominantly human-origin genomes, direct evidence of environmental transmission, such as patient exposure history or matched animal and environmental samples, was not available here [12]. Retrospective studies have reclassified historical human *S. suis* infections in South America as *S. parasuis*, suggesting that the geographic distribution of this organism may be underestimated [35]. The potential for environmental exposure, together with the ability of *S. parasuis* to acquire and disseminate AMR genes through mobile genetic elements, supports a cross-sectoral One Health surveillance approach [36]. Priorities include improved MALDI-TOF MS reference databases, validated species-specific molecular diagnostics, and linked human, animal, food, and environmental sampling to clarify transmission routes.

## 4. Materials and Methods

### 4.1. Bacterial Isolation and Preliminary Identification

A pleural fluid specimen was collected from a patient with pneumonia at Guangdong Provincial People’s Hospital (April 2025). The specimen was cultured on 5% sheep blood agar at 37 °C with 5% CO_2_ for 18–24 h. Suspected colonies were purified by repeated single-colony streaking and examined by Gram staining and light microscopy. A single colony from the morphologically uniform purified culture was selected for DNA extraction and whole-genome sequencing. Preliminary species-level identification was performed with VITEK MS (bioMérieux, Marcy-l’Étoile, France).

### 4.2. Antimicrobial Susceptibility Testing

MICs were determined using the VITEK 2 Compact system with an AST-ST03 card (bioMérieux, Marcy-l’Étoile, France; lot 5423493503). The antimicrobial panel was determined by the fixed configuration of the AST-ST03 card. *Streptococcus pneumoniae* ATCC 49619 was tested in parallel as the quality-control strain, and all observed MICs were within the expected ranges supplied for the AST-ST03 test system (Table 1). Because species-specific clinical breakpoints are unavailable for *S. parasuis*, the instrument-derived MICs were compared provisionally with CLSI M100 (36th ed., 2026) MIC criteria for viridans group streptococci (VGS) where an applicable criterion was available [20]. This pragmatic surrogate approach has been used in previous *S. parasuis* studies [10,11] and does not imply that *S. parasuis* belongs taxonomically to the viridans group. For trimethoprim–sulfamethoxazole, *S. pneumoniae* criteria were used as a surrogate. Teicoplanin, tigecycline, and moxifloxacin were reported as not interpreted because no applicable CLSI VGS MIC criterion was available. The antibiotics tested were penicillin, ampicillin, cefotaxime, ceftriaxone, vancomycin, teicoplanin, chloramphenicol, trimethoprim–sulfamethoxazole, erythromycin, tetracycline, tigecycline, levofloxacin, moxifloxacin, linezolid, and clindamycin.

### 4.3. Whole-Genome Sequencing, Hybrid Assembly, and Annotation

Genomic DNA was extracted with a bacterial DNA extraction kit (Omega Bio-tek, Norcross, GA, USA). Short-read sequencing (DNBSEQ-T7, BGI, Shenzhen, China) and long-read sequencing (Oxford Nanopore Technologies, Oxford, UK) were performed. Hybrid assembly was carried out with Unicycler v0.5.0 [37], followed by polishing with Pilon v1.24 [38]. Genome completeness and contamination were assessed with CheckM v1.2.2 [39]. Genome annotation was performed with Bakta v1.9.4 [40].

### 4.4. Genome-Based Species Identification and Sequence Typing

Species identity was first assessed with Mash v2.3 [41]. For confirmation, all available *S. parasuis* genomes in the NCBI database (accessed 13 July 2026) were filtered for quality (N50 ≥ 20 kb, completeness ≥ 95%, contamination ≤ 5%). ANI values were calculated with FastANI v1.3 [42], visualized in R, and reported in full in Supplementary Table S1. MLST was performed using the *S. parasuis* scheme at PubMLST [43]. The allelic profile and ST were assigned accordingly. A minimum spanning tree was generated with GrapeTree [44].

### 4.5. Core-Genome Phylogeny and Epidemiological Characteristics

Core-genome SNPs were identified with Snippy v4.6.0 (reference genome: SUT-286) [45] and filtered for recombination with Gubbins v3.2.1 [46]. A maximum-likelihood phylogenetic tree was constructed with IQ-TREE v2.4.0 using 1000 bootstrap replicates [47] and visualized with Interactive Tree Of Life (iTOL) v6 [48]. Predicted AMR genes were identified by querying the genome against the Comprehensive Antibiotic Resistance Database (CARD) [49] with ABRicate [50].

### 4.6. Pan-Genome Analysis and Source-Associated pan-GWAS

The pan-genome of HUGSPH and the selected reference genomes was analyzed with Roary v3.13.0 [51], using Bakta v1.9.4 annotations [40] as input. Core genes were defined at a 99% frequency threshold. The resulting gene presence–absence matrix was used to characterize shared and variable genomic components. Genomic similarity among isolates was visualized with roary plots [51].

To identify host-associated genetic determinants, we compared 7 human-origin and 9 swine-origin *S. parasuis* isolates. A Venn diagram was generated with the R package VennDiagram [52] to visualize the shared and host-specific gene repertoires. A pan-GWAS was conducted with Scoary v1.6.16 [53], with host origin as the binary phenotype. Phylogenetic correction was applied using a core-genome SNP-based maximum-likelihood tree generated with FastTree v2.1 [54], supplied to Scoary with the --newicktree option; 10,000 permutations were used. Association between each gene and host origin was first evaluated with Fisher’s exact test. *p* values were adjusted with the Benjamini–Hochberg procedure to control the false discovery rate (FDR). Genes with an FDR-adjusted *q* < 0.05 were considered significant. To ensure robustness, we then prioritized genes showing 100% presence in one host group and complete absence in the other.

### 4.7. Comparative Genomic and Functional Analysis

A circular whole-genome comparison was generated with BRIG v0.95 [55], using HUGSPH as the central reference. Pairwise core-genome SNP distances among human-origin isolates were calculated from the core-genome alignment with snp-dists v0.8 [56].

The Roary-derived pan-genome matrix was functionally annotated against the Clusters of Orthologous Genes (COG) database [57]. Principal coordinate analysis (PCoA) was performed in R v4.5.0 using Jaccard distances. HUGSPH-unique genes were functionally annotated with eggNOG-mapper v2 [58] against the Kyoto Encyclopedia of Genes and Genomes (KEGG) database [59]; KEGG Orthology (KO) assignments were mapped to pathways and summarized with a horizontal bar plot.

### 4.8. Virulence Gene Profiling and Galleria mellonella Infection Model

Predicted virulence-associated genes were identified by comparison with the Virulence Factor Database using VFanalyzer [60]. Healthy, active third-instar *Galleria mellonella* larvae (approximately 25 mm long and 300 mg; Shanghai Winnerbio Technology Co., Ltd., Shanghai, China) were starved for 24 h before infection and acclimated at 37 °C. HUGSPH cells were harvested, washed twice with sterile PBS, and resuspended in PBS. Suspensions were adjusted to deliver 1 × 10^4^, 1 × 10^5^, 1 × 10^6^, or 1 × 10^7^ CFU in a 10 µL inoculum. Larvae were randomized into groups of 10. Each larva was injected with 10 µL into the left posterior second proleg using a 0.26 mm-diameter, 9 mm-long needle. Control groups received 10 µL of sterile PBS or no injection. After inoculation, larvae were maintained at 37 °C in the dark and assessed at 24, 48, 72, and 96 h. Larvae were considered dead when they were melanized, immobile, and unresponsive to touch. Survival curves were generated by the Kaplan–Meier method and compared using the log-rank (Mantel–Cox) test in Prism v10.6.1 (GraphPad Software, Boston, MA, USA); *p* < 0.05 was considered statistically significant.

## 5. Conclusions

HUGSPH is a human pleural-fluid *S. parasuis* isolate assigned to the novel sequence type ST181 and positioned within a predominantly human-origin phylogenetic clade. Under explicitly stated surrogate CLSI criteria, its MICs were provisionally categorized as resistant to erythromycin, clindamycin, and levofloxacin, and its genome carried multiple predicted resistance-associated genes. Exploratory pan-GWAS identified source-associated gene-content patterns that may reflect lineage structure, while the *Galleria mellonella* model showed dose-dependent mortality under the tested conditions. These findings expand the genomic record of human clinical *S. parasuis* and support genome-based species confirmation and continued antimicrobial-resistance surveillance. Reference-method MIC testing, broader balanced isolate collections, comparator strains, targeted genetic experiments, and mammalian models are required to validate the susceptibility categories, host associations, and pathogenic mechanisms.

## Figures and Tables

**Table 1:** HUGSPH minimum inhibitory concentrations, provisional interpretations, and quality-control results.

Class	Drug	HUGSPH MIC (mg/L)	Provisional Interpretation	ATCC 49619 MIC (mg/L)	Expected QC Range (mg/L)
β-Lactams	Penicillin	≤0.06	S	0.25	0.25–1
β-Lactams	Cefotaxime	≤0.12	S	≤0.12	≤0.12
β-Lactams	Ceftriaxone	≤0.12	S	≤0.12	≤0.12
β-Lactams	Ampicillin	≤0.25	S	≤0.25	≤0.25
Glycopeptides	Vancomycin	≤0.12	S	≤0.12	≤0.12–0.25
Glycopeptides	Teicoplanin	≤0.12	NI	≤0.12	≤0.12
Phenicols	Chloramphenicol	4	S	2	2–8
Folate pathway antagonists	Trimethoprim–sulfamethoxazole	≤0.5/9.5 (≤10 total)	S	≤0.5/9.5 (≤10 total)	≤0.5/9.5–1/19 (≤10–20 total)
Macrolides	Erythromycin	2	R	≤0.12	≤0.12
Tetracyclines	Tetracycline	1	S	≤0.25	≤0.25–0.5
Tetracyclines	Tigecycline	≤0.06	NI	≤0.06	≤0.06–0.12
Fluoroquinolones	Levofloxacin	8	R	0.5	0.5–2
Fluoroquinolones	Moxifloxacin	0.25	NI	0.12	≤0.06–0.25
Oxazolidinones	Linezolid	≤2	S	≤2	≤2
Lincosamides	Clindamycin	1	R	≤0.25	≤0.25

**Note:** MIC, minimum inhibitory concentration; S, susceptible; R, resistant; NI, not interpreted; QC, quality control; VGS, viridans group streptococci. Because no species-specific clinical breakpoints are available for *S. parasuis*, categories are provisional. CLSI M100 (36th ed., 2026) VGS MIC criteria were used where applicable [20]. For trimethoprim–sulfamethoxazole, *S. pneumoniae* criteria were used as a surrogate, consistent with previous *S. parasuis* studies [10,11]. This surrogate use does not imply that *S. parasuis* belongs taxonomically to the VGS. Teicoplanin, tigecycline, and moxifloxacin were not interpreted because no applicable CLSI VGS MIC criteria were available. All observed ATCC 49619 MICs were within the expected AST-ST03 quality-control ranges supplied for the test system. Trimethoprim–sulfamethoxazole values are shown as trimethoprim/sulfamethoxazole concentrations, with total concentrations in parentheses.

**Table 2:** Exploratory pan-GWAS results for source-associated genes in *S. parasuis*.

Gene	Source Association	Human (*n* = 7)	Swine (*n* = 9)	FDR *q*-Value	Sensitivity (%)	Specificity (%)	Odds Ratio	Function/Description
*yxdM*	Human	7	0	0.0012	100	100	∞	ABC transporter permease protein YxdM
*nrdE2*	Human	7	0	0.0012	100	100	∞	Ribonucleoside-diphosphate reductase subunit alpha 2
*hcxA*	Human	7	0	0.0012	100	100	∞	Hydroxycarboxylate dehydrogenase A
*yfkJ*	Human	7	0	0.0012	100	100	∞	Low molecular weight protein-tyrosine-phosphatase YfkJ
*yodC*	Human	7	0	0.0012	100	100	∞	Putative NAD(P)H nitroreductase YodC
*nrdI*	Human	7	0	0.0012	100	100	∞	Catalyzes the conversion of ribonucleotides to the corresponding deoxyribonucleotides, providing precursors for DNA synthesis
*elaA*	Human	7	0	0.0012	100	100	∞	Putative N-acetyltransferase
*nrdF*	Human	7	0	0.0012	100	100	∞	Ribonucleoside-diphosphate reductase 2 subunit beta
*metQ*	Swine	0	9	0.0012	0	0	0	Putative D-methionine-binding lipoprotein MetQ
*metP*	Swine	0	9	0.0012	0	0	0	Methionine import system permease protein MetP
*metN*	Swine	0	9	0.0012	0	0	0	Methionine import ATP-binding protein MetN

**Note:** The comparison included seven human-origin and nine swine-origin genomes. These associations may reflect the phylogenetic structure of the sampled lineages and require validation in larger, balanced collections. FDR, false discovery rate.

**Table 3:** Predicted virulence-associated genes in HUGSPH.

Virulence-Factor Class	Predicted Factor	Related Gene(s)
Adherence	Agglutinin receptor	*orf00559*
Adherence	Choline-binding proteins	*cbpD*
Adherence	Fibronectin-binding proteins	*pavA*
Adherence	Sortase A	*srtA*
Adherence	Streptococcal plasmin receptor/GAPDH	*plr/gapA*
Adherence	Ebp pili	*orf01422*
Enzyme	Streptococcal enolase	*eno*
Immune evasion	Capsule	*cdsA*
Protease	Serine protease	*htrA*/*degP*
Protease	Trigger factor	*tig*/*ropA*
Cell surface components	Trehalose-recycling ABC transporter	*sugC*
Surface protein anchoring	Lipoprotein diacylglyceryl transferase	*lgt*

**Note:** Gene presence was predicted by comparison with the Virulence Factor Database and does not establish expression or functional contribution to virulence.

## Data Availability

Data supporting the findings are available from the corresponding authors upon reasonable request. The whole-genome assembly of *S. parasuis* strain HUGSPH is available in GenBank under accession number GCA_059553355.1. The multilocus sequence typing data and novel sequence type are available through PubMLST at https://pubmlst.org/organisms/streptococcus-parasuis (accessed on 8 September 2026).

## Supplementary Materials

The following supporting information can be downloaded at: https://www.mdpi.com/article/10.3390/antibiotics15090893/s1, Table S1. Pairwise average nucleotide identity matrix of HUGSPH and 39 reference *Streptococcus parasuis* genomes.

## Abbreviations

The following abbreviations are used in this manuscript:

AMR	Antimicrobial resistance
ANI	Average nucleotide identity
CARD	Comprehensive Antibiotic Resistance Database
CFU	Colony-forming units
CLSI	Clinical and Laboratory Standards Institute
COG	Clusters of Orthologous Genes
FDR	False discovery rate
ICE	Integrative and conjugative element
MALDI-TOF MS	Matrix-assisted laser desorption/ionization time-of-flight mass spectrometry
MCG	Minimum core genome
MIC	Minimum inhibitory concentration
MLST	Multilocus sequence typing
PCoA	Principal coordinate analysis
SNP	Single-nucleotide polymorphism
WGS	Whole-genome sequencing
